# Clinical and methodological factors affecting non-transferrin-bound iron values using a novel fluorescent bead assay

**DOI:** 10.1016/j.trsl.2016.05.005

**Published:** 2016-11

**Authors:** Maciej W. Garbowski, Yongmin Ma, Suthat Fucharoen, Somdet Srichairatanakool, Robert Hider, John B. Porter

**Affiliations:** a Research Haematology Department, Cancer Institute, University College London, UK; b University College London Hospitals, Haematology Department, London, UK; c Institute of Pharmaceutical Sciences, King's College London, London, UK; d College of Pharmaceutical Science, Zhejiang Chinese Medical University, Hangzhou, China; e Thalassemia Research Centre, Institute of Science and Technology for Research and Development, Mahidol University Hospital, Salaya, Nakhon Pathom, Thailand; f Department of Biochemistry, Faculty of Medicine, Chiang-Mai University Hospital, Chiangmai, Thailand

**Keywords:** AAS, atomic absorption standard, AlAT, alanine-aminotransferase, CFBs, control fluorescent beads, CI, confidence interval, CIC, cardiac iron content, CSA, congenital sideroblastic anemia, DBA, Diamodn-Blackfan anemia, DCI, directly chelatable iron, DFO, deferoxamine, DFP, deferiprone, DFX, deferasirox, ELISA, enzyme-linked immunosorbent assay, f.c., final concentration, FBC, full blood count, IQR, interquartile range, LIC, liver iron content, LPI, labile plasma iron, MDS, myelodysplastic syndrome, MOPS, 3-(N-morpholino)propanesulfonic acid, NRBC, nucleated red blood cells, NTA, nitrilotriacetic acid, NTBI, nontransferrin-bound iron, SD, standard deviation, SF, serum ferritin, sTfR, soluble transferrin receptors, Tf, transferrin, TfSat, transferrin saturation, ULN, upper limit of normal, UV, ultraviolet

## Abstract

Nontransferrin-bound iron (NTBI) is a heterogeneously speciated plasma iron, typically detectable when transferrin saturation (TfSat) exceeds 75%. Here, we examine factors affecting NTBI levels by a recently discovered direct chelator-based (CP851) fluorescent bead-linked flow-cytometric assay (bead-NTBI), compared with the established indirect nitrilotriacetate (NTA) assay in 122 iron-overloaded patients, including 64 on recent iron chelation therapy and 13 healthy volunteers. Both methods correlated (r = 0.57, *P* < 0.0001) but with low agreement, attributable to 2 major factors: (1) the NTA method, unlike the bead method, is highly dependent on TfSat, with NTBI under-estimation at low TfSat and over-estimation once Tf is saturated, (2) the bead method detects <3-fold higher values than the NTA assay in patients on recent deferiprone-containing chelation due to greater detection of chelate complexes but lower values for patients on deferasirox. The optimal timing of sample collection relative to chelation dosing requires further study. Patients with splenectomy, high-storage iron, and increased erythropoiesis had greater discrepancy between assays, consistent with differential access by both methods to the NTBI pools associated with these clinical variables. The bead-NTBI assay has advantages over the NTA assay, being less dependent on TfSat, hence of less tendency for false-negative or false-positive values at low and high TfSat, respectively.

**At a Glance Commentary**

Garbowski MW, et al.

**Background**

Non-transferrin-bound iron (NTBI) is increasingly understood as a multispeciated plasma iron pool, regulated separately from transferrin-bound iron and implicated in the complications of iron overload. The established nitrilotriacetate (NTA)-NTBI method is not optimal for distinguishing transferrin bound iron from NTBI.

**Translational Significance**

Here, we compared a novel fluorescent bead method with the NTA method, across clinical diagnoses. The NTA assay underestimates or overestimates NTBI at low- or high-transferrin saturations respectively, which the bead assay being robust to effects of transferrin does not. The greater specificity of the bead assay should clarify links between raised NTBI levels and their clinical consequences.

## Introduction

Plasma non-transferrin-bound iron (NTBI), first described in 1978,^1^ is a pathological iron pool detectable when Tf saturation exceeds 75%.^2^, ^3^, ^4^ NTBI appears when iron influx into the plasma compartment exceeds iron efflux, for example, with iron overload, ineffective erythropoiesis, or decreased transferrin iron clearance in erythroid hypoplasia.^5^ NTBI is considered the main conduit of hepatic^6^, ^7^, ^8^ and extra-hepatic^9^, ^10^, ^11^, ^12^ iron loading of tissues, under hemosiderotic conditions. Quantitating NTBI is of value in understanding NTBI generation under different pathophysiological settings^5^ but can also be potentially useful in the management of iron-overloaded patients. However, using established methods for NTBI quantitation, clear consensus, and guidelines on how to use NTBI measurement in patient management have yet to emerge. This is partly because NTBI is multispeciated, consisting of a range of iron-citrate,^13^, ^14^ albumin-bound complexes,^13^ glycated protein-iron complexes,^15^, ^16^ or iron-chelate complexes in recently chelated patients.^17^ Consequently, it is unlikely that NTBI assays relying on different principles will measure different NTBI species to the same extent. Hence, a consistent pattern of association between NTBI values and clinical outcomes has yet to emerge.

There is, therefore, a need to identify a robust and well-characterized NTBI assay that can be applied in a standardized manner in the management of iron-overloaded patients. A range of NTBI methods used previously differ considerably in their detection principles and reported reference ranges.^18^ The most long-standing and frequently reported NTBI method^19^ involves iron capture from NTBI by a high concentration of a low affinity/specificity iron chelator, nitrilotriacetic acid (NTA, 80 mM), followed by ultrafiltration and detection of NTA-iron by high-performance liquid chromatography^2^, ^19^ or spectrophotometrically.^3^ Another approach is measuring NTBI indirectly by quantifying the redox-active subset of NTBI, which has been termed the ‘labile plasma iron’ assay.^20^ A further approach is measuring the directly chelatable iron with a fluorophore-labeled high-affinity chelator,^18^, ^21^, ^22^ but background fluorescence in plasma may interfere with data interpretation. Most recently, an adaptation of this approach was described, using a high-affinity fluorescent chelator CP851, covalently linked to magnetic beads with fluorescence signal separated flow-cytometrically from plasma autofluorescence.^23^ This potentially circumvents the auto-fluorescence problem in the plasma sample and problems related to the indirect capture of NTBI by NTA.

The initial paper describing the bead method^23^ examined only 30 patients and did not, therefore, explore the variables affecting the agreement between the NTA and the bead method systematically. In particular, the effects of TfSat, chelators, splenectomy status, and underlying diagnosis were not explored. A recent round robin^18^ comparing various NTBI and labile plasma iron methods on 60 patients reported their overall lack of agreement in absolute values despite similar correlations but did not specifically look at the agreement between these 2 methods and could not, therefore, comment on the possible reasons for their poor agreement. Here, we compare levels of NTBI detected by this assay with the NTA method in various clinical conditions, including 122 iron-overloaded patients with approximately half (n = 64) receiving regular chelation therapy. Part of this work was presented as Abstract no. 241 at BioIron Conference Sep 6-10, 2015 in Zhejiang University, China.

## Materials and Methods

### Patients

One hundred twenty-two clinical blood samples from iron-overloaded patients and 13 healthy volunteers, obtained from 3 hospitals (affiliations 2, 4, and 5), were analyzed using the CP851-NTBI assay and NTA-NTBI assay. Diagnoses included are listed in Table I. Ethical approval was obtained for the study at the respective institutions where blood samples were collected, and patients signed informed consent forms before sample collection.

### NTBI assays

#### NTA-NTBI assay

The NTA-NTBI method previously described^19^ was adopted with minor modifications. Briefly, 0.02 mL of 800-mM NTA (at pH = 7) was added to 0.18-mL serum and allowed to stand for 30 minutes at 22^°^C. The solution was ultrafiltered using Whatman Vectaspin ultracentrifugation devices (30 kDa) at 12320*g* and the ultrafiltrate (0.02 mL) injected directly onto an high-performance liquid chromatography column (ChromSpher-ODS, 5 μM, 100 × 3mm, glass column fitted with an appropriate guard column) equilibrated with 5% acetonitrile and 3-mM deferiprone (DFP) in 5-mM MOPS (pH = 7.8). The NTA-iron complex then exchanges to form the DFP-iron complex detected at 460 nm by a Waters 996 photodiode array. Injecting standard concentrations of iron prepared in 80-mM NTA generated a standard curve. The 800-mM NTA solution used to treat the samples and prepare the standards is treated with 2-μM iron to normalize the background iron that contaminates reagents. This means that the zero standard gives a positive signal because it contains the added background iron as an NTA-complex. When unsaturated transferrin is present in sera, this additional background iron can be donated to vacant transferrin sites resulting in a loss of the background signal and yielding a negative NTBI value.

#### CP851 bead-NTBI assay

The standards for this assay were prepared as follows: 1-mM iron-NTA complex (1:2.5 molar ratio), prepared from 100-mM NTA and 18-mM atomic absorption standard iron solution, was diluted with MilliQ water to a final concentration (f.c.) between 0 and 100 μM. For the standard curve, 120 μL quantities of probe-labeled bead suspensions were incubated with 20 μL of buffered NTA-iron solutions of known concentration for 20 minutes at room temperature, with subsequent addition of 20 μL normal control serum (without free iron) and 40-μL paraformaldehyde (10% in MOPS) at 2% f.c. The suspensions in sealed 96-well plates were incubated at 37^°^C for 16 hours with shaking before fluorescence measurement by flow-cytometry. For serum samples of unknown iron concentrations, 140 μL quantities of beads were incubated with 20 μL of serum samples for 20 minutes, with subsequent addition of 40-μL paraformaldehyde at 2% (f.c.). In this study, we used chelatable fluorescent beads with normal human mixed serum as a control to set up the fluorescence at 100% and the relative fluorescence of chelatable fluorescent beads with patient serum was calculated accordingly. Measurements were carried out on Beckman Coulter FC500 flow-cytometer and analysis on Cell-Quest and FlowJo software. Gates were based on dot-plots of untreated bead populations. Median fluorescence of 10,000 events was recorded and corrected for bead auto-fluorescence. A standard curve was fitted with variable-slope sigmoidal dose-response function.

### Transferrin saturation

TfSat was determined by the urea-gel method^24^ with band quantitation using Scion Image software, normal reference range 16–56% (mean 36%).

### Routine blood test results and standard of medical care monitoring

Hematology tests: full blood count and red cell indices, reticulocytes, nucleated red blood cell, serum alanine transaminase, bilirubin, and ferritin were performed routinely in hospital laboratories; sTfR was measured using ELISA (R&D Systems).

Liver iron content was obtained from liver T2*^25^ or liver R2 Ferriscan,^26^ and cardiac iron content obtained from cardiac T2*.^27^ Medical records review provided information about chelation therapy, transfusion, and splenectomy status.

### Statistics

The data were presented descriptively using mean ± standard deviation (SD) or median ± interquartile range where appropriate, differences between subgroups were calculated using Wilcoxon test or paired *t*-test, dependent on distribution assumptions. Ninety-five percent confidence interval follows slope and Spearman or Pearson correlation coefficient value in brackets. Graphpad Prism, Ver. 6.0 plots were used for slope comparison and Bland-Altman plots^28^ to illustrate agreement between methods. A *P* < 0.05 was deemed statistically significant.

## Results

Correlations, distributions, and agreement between NTA-NTBI and bead-NTBI were first examined. The TfSat effects on measured values were compared, followed by the effects of chelation therapy, erythropoiesis, storage iron, and splenectomy status on NTBI measured by both methods.

### Correlation and agreement of the 2 assays in all samples

**Fig 1:**
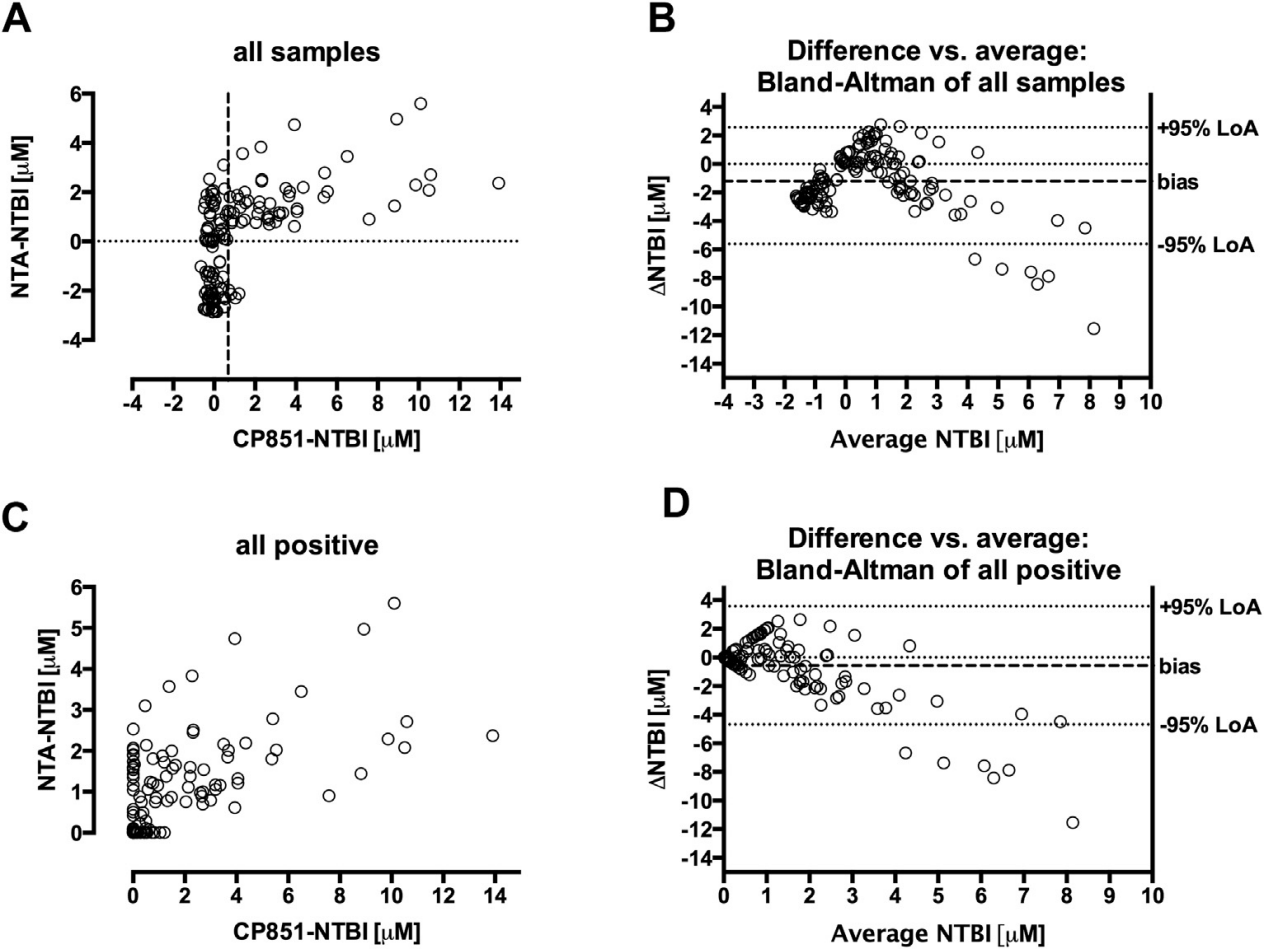
Comparison of NTA and beads method for NTBI measurement. (**A**) NTA and beads (CP851) methods plotted for all samples using the original scale. Correlation coefficient r = 0.57 (0.44–0.68) *P* < 0.0001 (**B**) Agreement shown using Bland-Altman analysis of difference (ΔNTBI = NTA-CP851) vs. mean of the 2 methods on data from panel A with bias −1.21 ± 2.25 μM and 95% LoA (limits of agreement) from −5.6 to 3.2 μM. (**C**) Both methods for NTBI measurement plotted as in panel A but with negative values by both methods shown as zero (absent NTBI) Correlation coefficient r = 0.6 (0.48–0.7) *P* < 0.0001. (**D**) Agreement shown using difference vs. mean Bland-Altman analysis of data in panel C, bias −0.56 ± 2.1 μM, 95% LoA −4.68 to 3.56 μM. NTBI, nontransferrin-bound iron; NTA, nitrilotriacetate.

There was a medium-strong correlation between bead-NTBI and NTA-NTBI for all samples (Fig 1*A*). Because negative values were obtained in the NTA assay, the graph was replotted treating all negative values as zero (Fig 1*C*). In Bland-Altman analysis (Fig 1*B* and *D*), the differences between methods notably increased at mean NTBI>2.5 μM being even greater above mean values of 4 μM. For mean values between <1 and 4 μM, the bias is not constant, being negative at <0 μM (NTA method less than bead method) and positive between 0 and 2 μM (bead higher than NTA). Treating negative values as zero does not improve agreement (Fig 1*D*), suggesting that negative values are not the main reason for the lack of agreement.

### The distribution of NTBI values by the 2 methods

**Fig 2:**
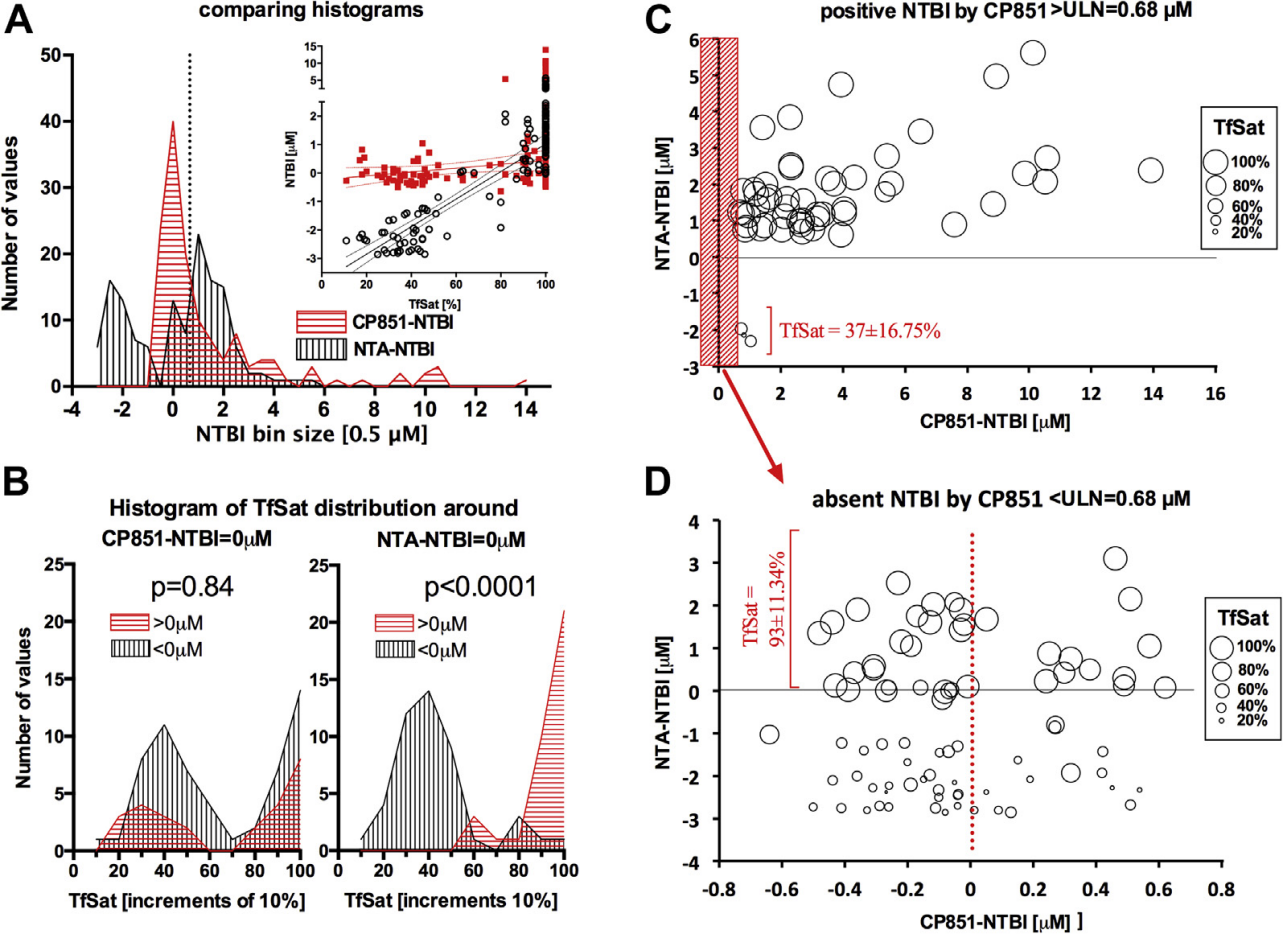
Distribution of CP851-NTBI, NTA-NTBI, and transferrin saturation values with analysis of false-positive and false-negative NTA-NTBI values. (**A**) Comparing frequency distribution histograms of CP851-NTBI (red) and NTA-NTBI (black) in 135 pairs; frequency (number of values) of NTBI per interval of 0.5 μM (bin size) is shown. **Inset** shows the relationship between NTBI level and urea-gel TfSat for both methods: CP851-NTBI (red) and NTA-NTBI (black); best-fit linear regression slope differences (*P* < 0.0001) with 100% TfSat points excluded from regression. (**B**) Plots of Tf saturation on x-axis (in 10% increments, bin center) against frequency of TfSat observations (y-axis) for NTBI values < or > 0 μM. The left panel shows bead method and right panel NTA method. The TfSat distributions for NTBI values > 0 μM are significantly different from those under 0 μM by the NTA method only (Mann-Whitney test); at low TfSat negative NTBI values are significantly more likely by NTA method and at high TfSat positive NTBI values are more likely by NTA method. These differences are not apparent by the beads method. (**C**) The plot of NTA-NTBI values vs. true-positive bead-NTBI values (above ULN = 0.68 μM—dashed line in Figure 1A), the size of the point reflects TfSat. Data in the red-shaded box (below ULN) are shown in panel D. (**D**) The plot of NTA-NTBI vs. true-negative bead-NTBI (red-shaded box in panel C corresponds to the data to the left of the dashed line in Figure 1*A*) with point size reflecting TfSat. Mean TfSat for NTA values >0 μM was 93%, 19/36 were 100%, range 62–100%, median, 25th and 75th percentile: 100, 91, 100%. Mean TfSat for NTA values <0 μM was 41.5%, 1/46 was 100%, range 11–100%, median, 25th and 75th percentile: 39.5, 31.5, 46%. NTBI, nontransferrin-bound iron; NTA, nitrilotriacetate; TfSat, transferrin saturation. (For interpretation of the references to color in this figure legend, the reader is referred to the Web version of this article.)

The NTBI distributions for both methods differ considerably between the assays (Fig 2*A*). With the NTA-NTBI (black) there is a clear population of negative values that is absent with the bead-NTBI (red). There is then a second population of positive NTA values (mode = 1.5 μM) that is absent with the bead-NTBI where the mode = 0 μM. Finally, there is a long-positive tail of high-bead-NTBI up to 14 μM that is absent with the NTA method. The further analysis below aims to determine the meaning of these distributional differences.

### Relationship of TfSat to NTBI values by the 2 methods

#### NTBI values are related to TfSat by NTA assay but not bead assay

The relationship of NTBI values by both methods to TfSat is presented in the inset of Fig 2*A*. TfSat has a strong relationship to the NTA-NTBI over its negative range, but not to the bead-NTBI. In particular, the NTA-NTBI values fall with decreasing TfSat, unlike with the bead assay, with the NTA-NTBI becoming negative for TfSat<80%, but no such effect is seen with the bead method, which confirms previously published results on a smaller group of patients.^18^ Because of the relationship of NTBI to TfSat by the NTA method and not the bead method, we examined the distribution of TfSat shown in Fig 2*B* for the bead-NTBI (left) and NTA-NTBI (right) for samples where positive (red) or negative (black) NTBI values were obtained. It appears that whereas with the bead-NTBI TfSat has a similar (*P* = 0.84) distribution for patients with positive or negative NTBI values, by contrast, the negative NTA-NTBI are largely confined to patients with TfSat<60% and the positive values to patients with TfSat>80% (*P* < 0.0001). This supports the relevance of low TfSat to negative NTA-NTBI but not bead-NTBI values.

#### Relevance of high TfSat to false-positive values with NTA assay

‘True-negative’ bead-NTBI values (below upper limit of normal [ULN] = 0.68 μM i.e., 3×SD above normal control mean = −0.1 μM, SD = 0.26 μM, marked as dashed line in Fig 1*A*) were plotted against NTA-NTBI with the point size reflecting TfSat (Fig 2*D*, note Fig 2*C* and *D* are derived from Fig 1*A*). Positive NTA-NTBI associates exclusively with high TfSat, whereas negative NTA-NTBI almost exclusively with low TfSat (93 ± 11.3% vs. 41.5 ± 18.2%, *P* < 0.0001). This means that the positive values by the NTA method that are negative by the bead method associate with high TfSat and are ‘false-positive’. This is consistent with iron being stripped off holotransferrin by NTA and not by CP851 (discussed). In contrast, no such distributional TfSat differences were seen between the negative and positive bead-NTBI values (on both sides of the red dotted line, Fig 2*B* and *D*). It is also possible that for other NTA-NTBI assays (N3 in the round robin,^18^ or using Co or Mn blocking^29^) the relationship with the TfSat may be different.

#### Relevance of low TfSat to false-negative values with NTA assay

The NTA-NTBI values were plotted against ‘true-positive’ bead-NTBI values (above ULN, Fig 2*C*, mark the range change on the x-axis in Fig 2*C* vs. 2D) with the point size reflecting TfSat. It appears that the ‘true-positive’ bead-NTBI is also typically positive by the NTA method only when associated with high TfSat. In the small number of values where the NTA-NTBI is negative, TfSat is low. Thus ‘false-negative’ NTA-NTBI values occur only in the presence of apotransferrin.

### Relationship of chelation therapy and NTBI values by both methods

**Fig 3:**
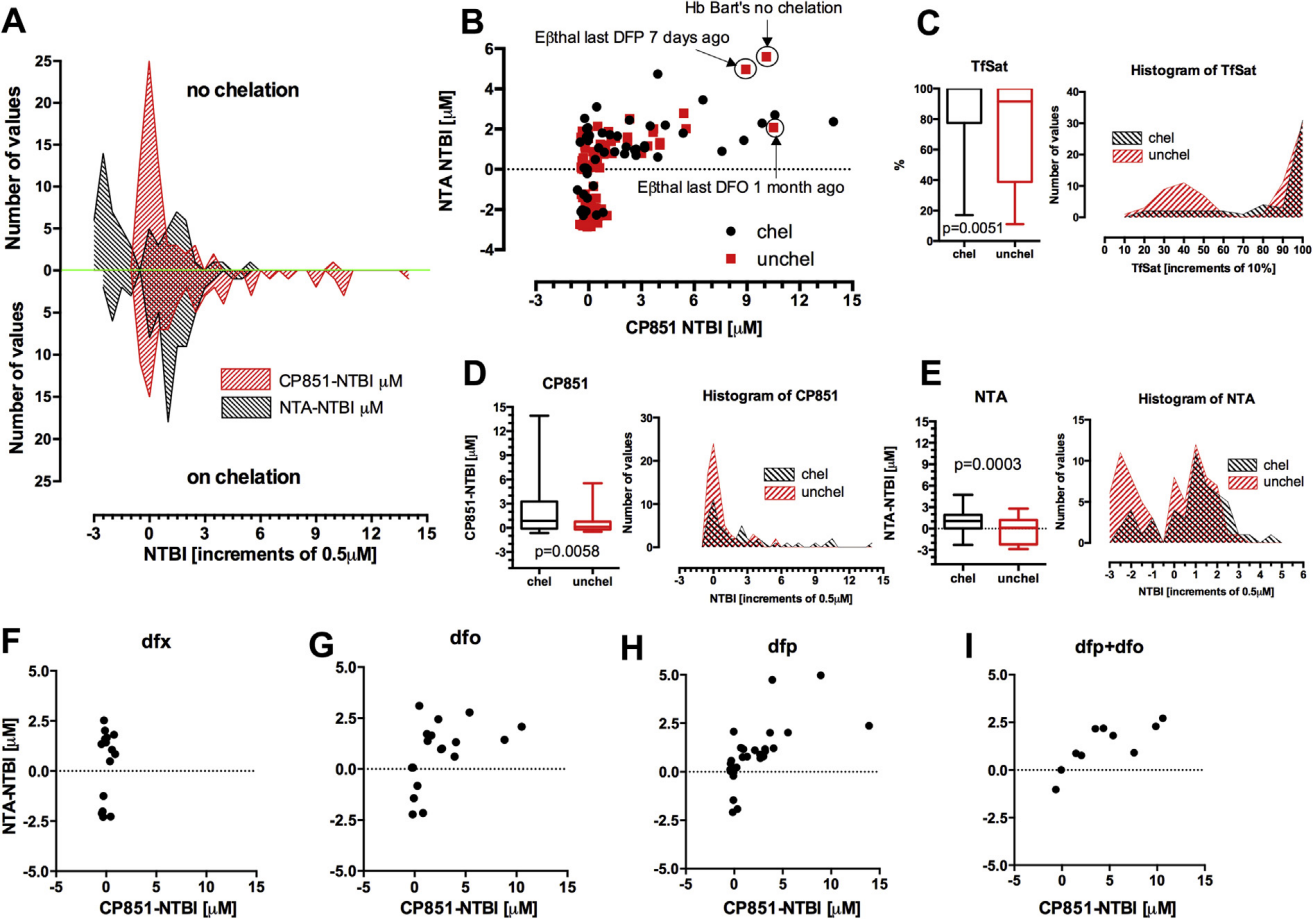
Effect of chelation on method agreement. (**A**). Comparison of NTBI distributions in patients not chelated (upper panel) and chelated (lower panel), red symbols show the bead CP851 assay and black symbols the NTA assay. (**B**) Comparison of NTA method vs. bead method in patients with (black) and without (red) chelation, of the outliers marked only the chelation ones were excluded from analysis in panels (**C**–**E**). (**C**) Comparison of TfSat distributions in chelated and unchelated patients seen in B, Mann–Whitney test. (**D**) Comparison of CP851-NTBI in chelated and unchelated patients seen in B, Mann–Whitney test (**E**) Comparison of NTA-NTBI in chelated and unchelated patients seen in B, Mann–Whitney test. (**F**) NTA method vs bead method in patients on deferasirox, no correlation (ns) (**G**) NTA method vs bead method in patients on desferrioxamine, correlation coefficient r = 0.47 (0.01–0.77), *P* = 0.05. (**H**) NTA method vs bead method in patients on deferiprone, correlation coefficient r = 0.63 (0.34–0.81), *P* = 0.0003. (**I**) NTA method vs bead method in patients on combination therapy of deferiprone and desferrioxamine, correlation coefficient r = 0.78 (0.3–0.95), *P* = 0.0076. NTBI, nontransferrin-bound iron; NTA, nitrilotriacetate; TfSat, transferrin saturation. (For interpretation of the references to color in this figure legend, the reader is referred to the Web version of this article.)

The presence of iron-chelate complexes or iron-free chelator could in principle influence the assay behavior *in vitro*.^17^, ^23^, ^30^ We examined these potential effects by comparing values in regularly chelated patients with those not receiving chelation. Both NTBI assays are plotted for chelated and unchelated patients in Fig 3*A*. The range of values differs in the upper (no chelation) and lower panels (recent chelation), particularly for the bead-NTBI (red), where there is a ‘tail’ of high values (5–15 μM) in chelated patients (Fig 3*B* and *D*). In principle, this could represent iron-chelate complexes detected as bead-NTBI. With the NTA-NTBI (black), the difference between chelated and unchelated patients is subtler, but it appears that the proportion of patients with negative values decreases, whereas that of patients with slightly positive values (up to 2 μM) increases on chelation (Fig 3*B* and *E*). This could again represent the effects of iron-chelate complexes on the assays.

To investigate this in more detail, the values obtained with both assays were plotted for patients on different chelation regimens (Fig 3F–I). Notably, values obtained on the same samples with the bead assay (max. 15 μM) are about 3-fold higher than with the NTA assay (max. 5 μM) for deferiprone or combination (desferrioxamine + deferiprone) treated patients but not for deferasirox (DFX), where bead-NTBI values are actually lower than NTA-NTBI. Patients receiving desferrioxamine usually show about 2 to 3-fold higher values with the bead assay than the NTA-NTBI. NTBI levels correlated within the desferrioxamine (0.47 (0.01, 0.77), *P* = 0.05), and the deferiprone (0.62, (0.34, 0.81) *P* = 0.0003) groups, but without agreement of absolute values, whereas in the DFX patients correlation was absent.

Differences in patient sampling on DFX and deferiprone offer a partial explanation. All patients receiving DFX had a 72-hour chelation washout, strictly observed before sampling, making the presence of the contaminating iron-DFX complexes unlikely. By contrast, in most of the deferiprone-treated patient samples, where 72-hour washout was not observed, the iron-chelate complexes would be expected to be present.^17^, ^23^ It appears that the complexes of deferiprone are more readily detectable as bead-NTBI than NTA-NTBI but different incubation times within the assay procedure are probably partly responsible for it. The detection of high levels by the bead assay in 2 patients on desferrioxamine (Fig 3*G*) is not predicted from in vitro evaluation of the bead assay^23^ and is difficult to explain unless patients were taking deferiprone.

### Other factors affecting relationships between assays

**Fig S1:**
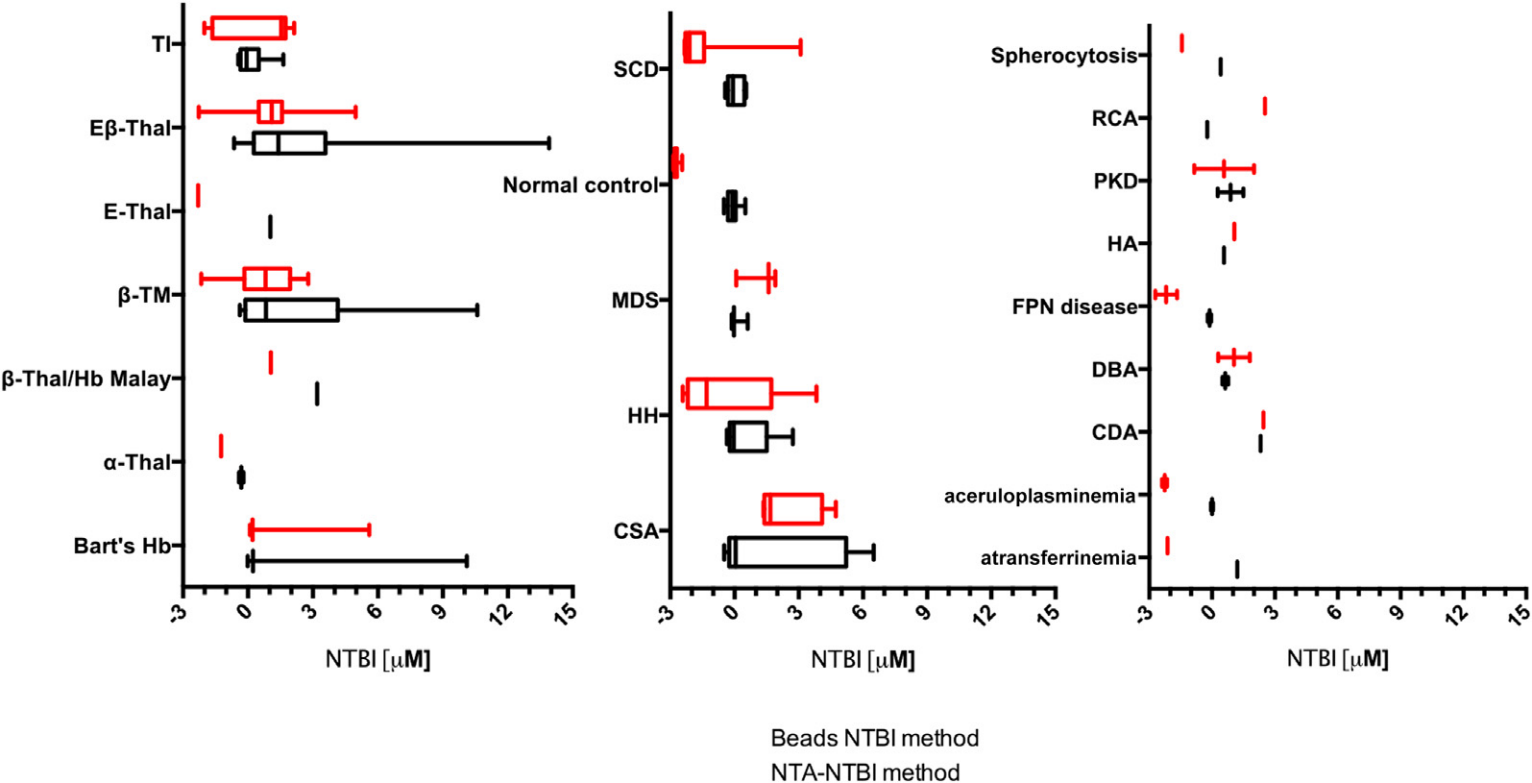
Box and whisker plot (range) of NTBI in healthy controls and various conditions of iron overload using a standard analytical method (NTA-NTBI) and the proposed bead-based NTBI method. NTA, nitrilotriacetate; NTBI, nontransferrin-bound iron.

**Fig S2:**
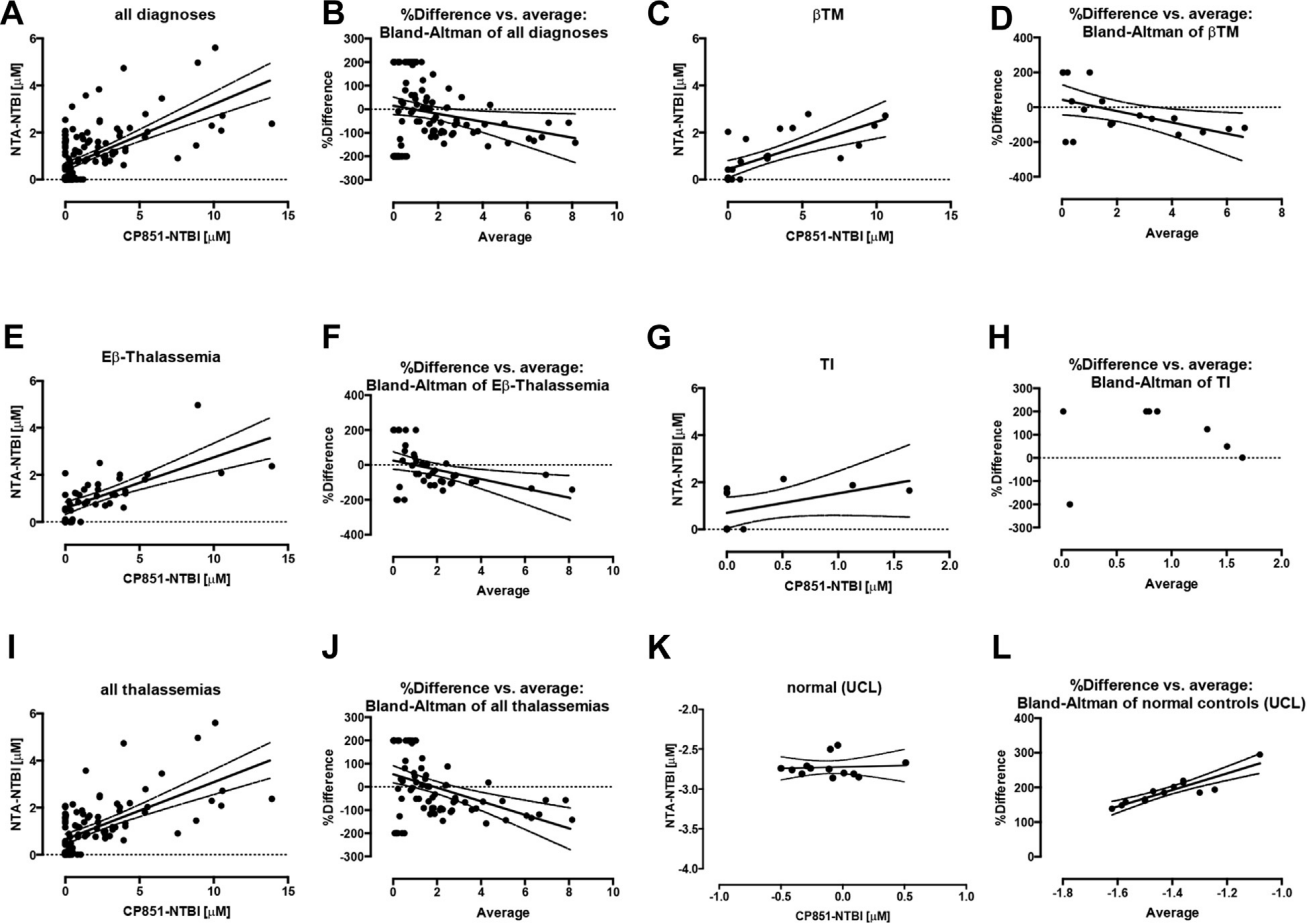
Comparison and Bland-Altman analysis of agreement of NTA and CP851-NTBI methods in different diagnosis groups. Negative values by both methods have been treated as zero. (**A**-**B**) all diagnoses; slope *P* < 0.0001. (**C**-**D**) beta-thalassemia major; slope *P* < 0.0001. (**E**-**F**) E-beta-thalassemia; slope *P* < 0.0001. (**G-H**) thalassemia intermedia; slope not significant. (**I**-**J**) all thalassemias; slope *P* < 0.0001. (**K**-**L**) normal volunteers; slope not significant. All Bland-Altman analyses show lack of acceptable agreement except for the normal samples where the low-scatter systematic bias could be corrected for using the formula (239.1 ± 35.12[%]*avgNTBI[uM]+527.3 ± 50[%]) (27). NTBI, nontransferrin-bound iron; NTA, nitrilotriacetate.

**Fig S3:**
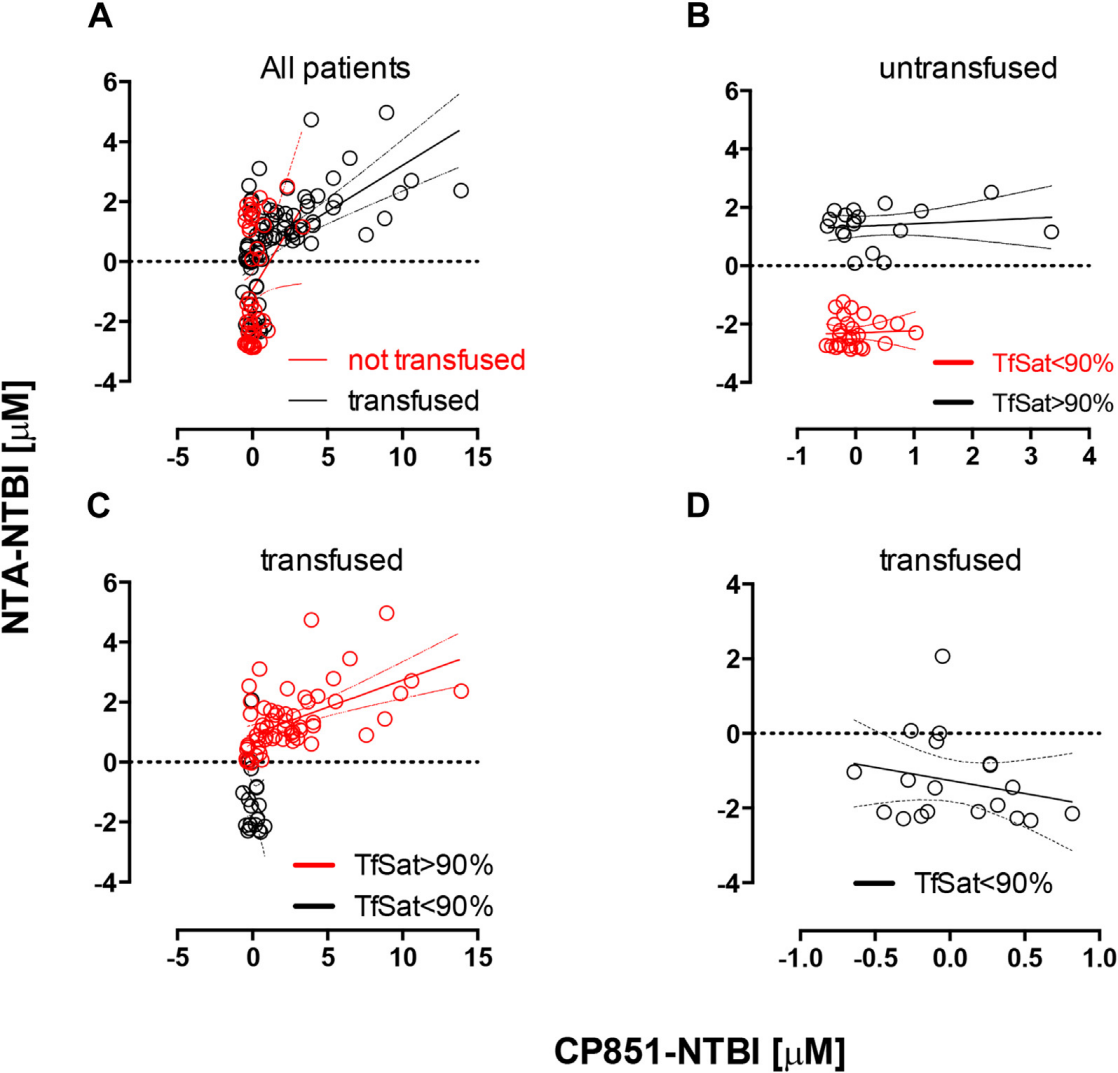
The effect of transferrin saturation on method agreement in transfused and untransfused patients. (**A**) Plot of both NTBI methods in transfused (black) and untransfused patients (red). (**B**) Plot of both NTBI methods in untransfused patients (red in panel A). Samples with TfSat>90% are now shown in red and those with TfSat<90% in black. (**C**) Both NTBI methods plotted in transfused patients (black in panel A), here further grouped according to TfSat in sample: with significant slope in TfSat>90% (red, *P* < 0.0001) and insignificant in TfSat<90% (black). (**D**) Both methods plotted for transfused patients with TfSat <90% show insignificant slope (black symbols from panel E magnified). TfSat, transferrin saturation; NTBI, nontransferrin-bound iron.

**Fig S4:**
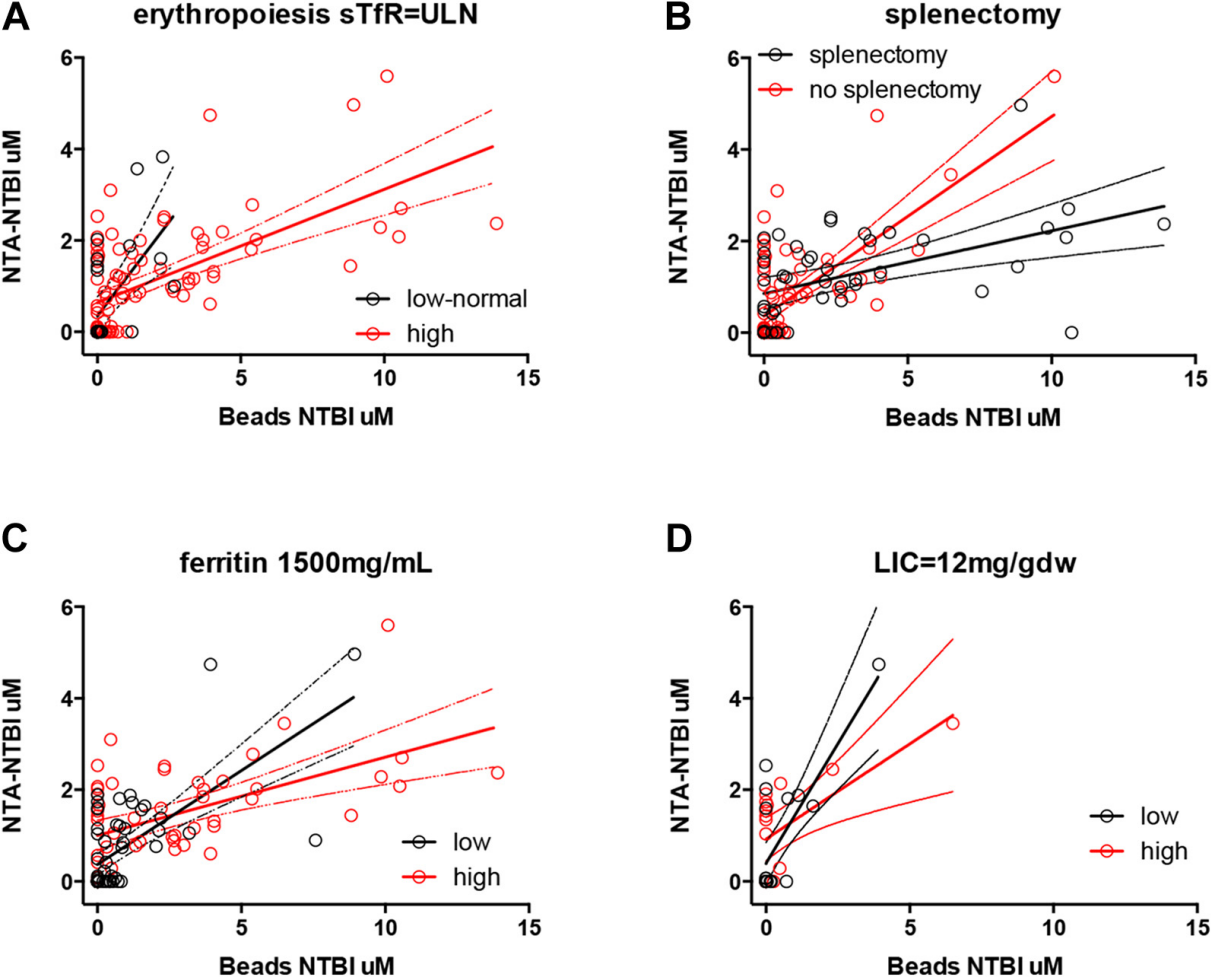
Comparison of NTA-NTBI and CP851-NTBI methods in groups with different erythropoietic, spleen, ferritin, and LIC status. (**A**) Slope comparison between high- and low-normal erythropoietic status defined by soluble transferrin receptor: both slopes are significant (0.25 ± 0.03, *P* < 0.0001 and 0.82 ± 0.22, *P* = 0.001) and different (*P* = 0.01); patients mean sTfR (n = 117) was 93.46 ± 68.1 nM (median 88.24, IQR 30.1–133.2, range 5.38–332.5; **B**) Slope comparison between patients with and without splenectomy: both slopes are significant (0.14 ± 0.04, *P* = 0.0005, and 0.44 ± 0.05, *P* < 0.0001) and different (*P* < 0.0001). (**C**) Slope comparison between low- and high-ferritin groups (1500μg/L): both slopes are significant (0.41 ± 0.06 and 0.17 ± 0.04, both *P* < 0.0001) and different (*P* < 0.0013). (**D**) Slope comparison in low- and high-LIC groups (12 mg/gdw): both slopes significant (1.05 ± 0.21, *P* = 0.0001 and 0.42 ± 0.13, *P* = 0.006) and different (*P* = 0.015). LIC, liver iron content; sTfR, soluble transferrin receptors; NTBI, nontransferrin-bound iron; NTA, nitrilotriacetate; IQR, interquartile range.

Using univariate analysis, we investigated the effects of diagnoses, transfusion status, erythropoiesis (by sTfR), splenectomy, and storage iron, on the levels of NTBI by both assays. There was no relationship between NTBI and diagnoses, but both NTBI methods were differentially affected by high-erythropoietic status, splenectomy, and high-iron storage (as detailed in Supplementary Material: Subgroup univariate analysis, Table SI, Fig S1, Fig S2, Fig S3, Fig S4).

### Multiple regression

Multiple linear regression models were built to test which predictors explain differences between the NTA-NTBI and the bead-NTBI. The same or additional predictors may resurface as relevant when the absolute difference (bias) between methods is modeled, and this was attempted as a control analysis. Furthermore, we have modeled transferrin saturation using the same set of potential predictors as for the other models (Table II). The bead-NTBI model explained 25% of the variability in NTBI using *TfSat*, *splenectomy*, and deferoxamine (*DFO*) + *DFP* as positive predictors. The NTA-NTBI model explained >75% of the variability in NTBI, with *TfSat*, *splenectomy* as positive, and *sTfR* as negative predictors. The bias model, that is, the difference CP851-NTBI less NTA-NTBI, was predicted negatively by *TfSat* and *DFX*, and positively by *splenectomy*, *DFO* + *DFP*, *chelation* (yes/no), and “*normal*”, in a model explaining 24% of the NTBI variability. The TfSat model explained 42% of the NTBI variability using *thalassemia*, *Eβ-thalassemia*, myelodysplastic syndrome, congenital sideroblastic anemia, Diamond-Blackfan anemia, and serum ferritin as positive predictors.

## Discussion

This study examined a range of iron-overloaded patients with and without iron chelation, which allowed comparison of the established NTA-based NTBI assay (NTA-NTBI) with a novel fluorescent bead-based assay (bead-NTBI) under a wide range of clinical conditions. Overall, although significant correlations exist between both methods, absolute values differ with wide 95% limits of agreement, consistent with the recent round-robin of NTBI assays.^18^ Here, by comparing values in a large number of iron-overloaded patients, we examine how both assays are differently affected by identifiable variables. These are relevant to the application and interpretation of both NTBI assays in specific patient populations. Two systematic differences between the assays have been identified. First, the NTA-NTBI is highly affected by TfSat, leading to under-estimations at low TfSat and over-estimation once transferrin is saturated. By contrast, the bead-NTBI is less dependent on either high or low TfSat. Second, while both assays give increased NTBI values in recently deferiprone-treated patients, due to detection of chelate-iron complexes, this effect is more pronounced with the bead-NTBI, leading to a further lack of agreement between the 2 assays.

Inspection of the NTBI distribution histograms for both assays, combined with the knowledge of the contrasting chelating properties of NTA and the hexadentate CP851 used in the bead method, provides insight into why the agreement is not high. With the NTA-NTBI, 2 major peaks are seen, first centering on −2.5 μM, and the second peak on 1.5 μM skewed rightward. With the bead-NTBI, these peaks are essentially absent with most low values clustered around zero and with a much more pronounced right skew for positive NTBI. These differences are consistent with the known properties of the ‘capture’ mechanisms of the assay chelators. Negative NTBI values obtained with the NTA method have been attributed to shuttling of iron present in 80-mM NTA onto apotransferrin during the initial incubation.^2^, ^31^, ^32^ This shuttling iron donation effect, due the greater stability of the hexadentate bead chelators, is absent in the bead method.^23^ Eighty-mM NTA not only donates chelated iron to iron-binding apotransferrin sites but also removes iron from holotransferrin in a time- and concentration-dependent manner,^3^ so that at 30 minutes 80-mM NTA mobilized 1–2% of transferrin iron (physiological concentration, TfSat = 50%). This represents 0.35–0.7-μM transferrin-bound iron potentially detected as ‘NTBI’ but could be as much as 1.4 μM with TfSat = 100% in our patients. Others noted similar effects.^33^, ^34^

The impact of such high-iron removal from transferrin has not been previously characterized, and here the comparison with the bead method, where iron is not stripped from holotransferrin (<0.2%) by the hexadentate chelator,^23^ demonstrates this effect more clearly. Given the robustness toward transferrin of the bead-assay, it can be used as a reference in an attempt to examine transferrin dependence of the NTA-method. Closer inspection of the normal and pathological bead-NTBI distributions suggests a cut-off above which values can be regarded as ‘true-positive’ and provides supportive evidence for the mechanisms underlying ‘false-positive’ and ‘false-negative’ values obtained with the NTA assay. With the bead-NTBI, values centring in the narrow dominant peak around the mode −0.04 μM (Fig 2*A*) are likely to represent an absence of true NTBI, given the comparable spread around the mean of the normal serum samples (−0.1 ± 0.26 μM with ULN of 0.68 μM mean+3SD, SD = 0.26 μM, Fig S2, Fig 2*K*). Bead-NTBI values > 0.68 μM are therefore likely to represent ‘true-positive’ NTBI. Clearly ‘false-negative’ values by the NTA method would then be those negative values obtained when the bead assay gives positive values > 0.68 μM. When this was checked, all the ‘false-negative’ NTA values occurred in samples with nonsaturated transferrin (45, 18, 48%, Fig 2*C*) where the predicted percentage of apotransferrin that can act as an acceptor for iron shuttled by NTA, approaches 30%, 65%, and 28%, respectively.^35^ By contrast, ‘false-positive’ NTA-NTBI would be those positive values that correspond to bead-NTBI<0.68 μM. All such ‘false-positive’ NTA values had highly saturated transferrin (mean 93%, range 62–100%) supporting the concept that such ‘false-positive’ NTBI values are obtained from the scavenging of iron from highly saturated transferrin.

The second factor, contributing to differences between both assays, is the effect of iron chelators or their iron complexes. With the NTA-NTBI, iron-free chelators present in plasma can act as acceptors for NTA-bound iron, potentially leading to the NTBI underestimation.^31^, ^32^ This donation can be blocked in the NTA assay by adding an excess of aluminum to samples before processing.^30^, ^32^ This is not a problem for the bead-NTBI, as the greater stability of the CP851-iron complex prevents iron donation to iron-free chelators in samples.^23^ The iron complexes of some chelators can also potentially interfere with the NTBI determination: with the NTA-method, the deferiprone-iron complexes are detected as ‘NTBI’ up to 1 week after drug cessation.^17^ In principle, the bead assay can also detect such complexes of deferiprone,^23^ and indeed our findings here suggest that these are detected to a greater extent than with the NTA assay (Fig 3G–I).

Owing to the high stability of ferrioxamine (1:1 iron(III):desferrioxamine), the bead-assay removes only a negligible amount of iron from DFO-iron complexes.^23^ Given this observation, it is difficult to explain the presence of 2 outliers in Fig 3*G*, and perhaps the iron-binding DFO metabolites should be considered. In the DFX-treated patients, NTBI is lower than in the other chelators using both assays but especially with the bead-assay, with values <1 μM (Fig 3*F*). This may reflect superior NTBI removal with DFX or that patients in this group had 72-hour washout from chelators, allowing full clearance of DFX-iron complexes. These complexes may clear more rapidly than those of deferiprone which are measurable >1 week after the last chelation dose.^17^ Thus in patients who are on regular chelation therapy, interpretation of NTBI values needs to be made concerning the timing of sampling, particularly with the bead-assay which has high affinity for iron-deferiprone and iron-DFX complexes.^23^

Clinical factors potentially affecting the detection levels of both NTBI assays were examined. Underlying diagnoses in themselves did not show significant differences between assays (Table II), but the extent of erythropoiesis did (Fig S4*A*): higher sTfR levels (and hence greater erythropoiesis and iron removal from transferrin) reduce NTA-NTBI, but not bead-NTBI values. As lower TfSat associates with decreased NTBI values due to NTA shuttling iron onto transferrin, clearance of transferrin-bound iron by enhanced erythropoiesis may increase iron shuttling by NTA and hence lower measured values. Negative prediction of the bias by TfSat suggests that the methods are differentially affected by TfSat, consistent with the methodological dependence of the NTA method on TfSat where NTA strips iron from transferrin, with NTBI overestimation rendering the bias lower. Likewise, DFX negatively predicts the bias because it affects only the bead-NTBI (Fig 3*F*), and this is a key issue we are currently investigating. Normal status increases the bias by rendering NTA values negative (ApoTf). Chelation increases the difference because it increases the bead-NTBI more than the NTA-NTBI when detecting deferiprone-iron complexes. Splenectomy predicts NTBI by either method using multivariate analysis and also associates with higher NTBI (by 0.5 or 1.3 μM, Table II). That splenectomy predicts bias is interesting while at the same time being a positive predictor of both methods separately because it implies that bead assay detects more NTBI in splenectomized patients rather than that the NTA method detects less (Fig S4*B*), suggesting it differentially affects the manner in which both methods may detect NTBI (splenectomy-dependent NTBI speciation differences). Higher NTA-NTBI in splenectomized TI patients was reported^36^ as was a greater risk of myocardial iron deposition after splenectomy.^37^ The mechanism for higher NTBI after splenectomy is unclear, but, we suggest, may relate to erythrocyte destruction being diverted from the spleen to the bone marrow. With relatively hypoxic bone marrow environment, oxidation from Fe^2+^ to Fe^3+^ will have slower kinetics, hence slower iron binding to transferrin, and a greater propensity to plasma NTBI formation. Similarly, higher levels of iron overload (serum ferritin or liver iron content) are associated with higher bead-NTBI than NTA-NTBI values, suggesting the bead method may detect some iron species associated with iron overload that are relatively unavailable for capture using the NTA method.

Unlike the NTBI values by either method, the TfSat is notably predicted by diagnoses and ferritin in our cohort. In the explanation of the ferritin effect on TfSat but not on NTBI (the latter also reported recently^18^), we distinguished plasma iron compartment into the transferrin part, changing dynamically below 100% (TBI), and into NTBI part, typically present when transferrin is 100% saturated (TfSat not changing dynamically anymore). This means that ferritin as a predictor of TfSat marks the independent effect of the degree of tissue iron overload on the changes in the transferrin part of plasma iron compartment (i.e., TfSat increases when ferritin increases), but such independent effect on the level of NTBI, once TfSat is saturated, is absent. In other words, if NTBI is considered in a continuum with TBI above the saturation point of Tf, it is evident that plasma iron above the saturation point of Tf (NTBI) does not associate with ferritin (and therefore with iron overload), whereas the plasma iron on transferrin does. This is very interesting to us because it may suggest that regulation of serum iron varies by compartment: NTBI is less dependent for its generation and persistence on iron overload per se, and likely more dependent on other factors such as those that determine its removal rather than generation (tissue uptake-erythroid, hepatic).^5^, ^38^ Finally, that sTfR does not predict TfSat but does negatively predict NTBI implies that the rate of transferrin off-loading in the marrow may affect the NTBI compartment without apotransferrin being detected peripherally.^38^

In conclusion, we have identified that the TfSat in the blood sample affects values obtained with the NTA assay to a greater extent than with the bead assay. This results from iron donation to apotransferrin by NTA and/or stripping of iron from saturated transferrin by NTA. Neither of these effects is significant with the bead assay, which may, therefore, be more specific for true NTBI determination. These findings are consistent with first principles, namely differential access to transferrin iron of both methods, but other mechanisms may be involved, which require further systematic study. The presence of iron-chelate complexes in patients on chelation, particularly with deferiprone, increases values obtained with the bead assay more than with the NTA assay. Recent chelation history (minimum a week before blood sampling) needs accounting for when interpreting NTBI values obtained with either method. Other differences between the assays regarding the effects of splenectomy, levels of iron overload and endogenous erythropoiesis are consistent with both assays differentially accessing NTBI pools that vary with these clinical variables. Future work will need to identify whether NTBI values obtained with the bead assay are more clinically predictive of trends, such as myocardial iron deposition than with the NTA assay. The effects of sample timing in relation to currently available chelation therapies need to be further defined with both assays. We recommend using both methods in NTBI research because the NTA-NTBI and the bead-NTBI do not detect exactly the same species of NTBI, and that further research is necessary to describe NTBI speciation in greater detail before a recommendation can be made (if at all) which method should the researchers rely on.

## Figures and Tables

**Table SI:** Subgroup analysis: correlations, slope comparison, and NTBI differences between transfusion, erythropoiesis, splenectomy, storage iron and chelator subgroups

Subgroup∗	Correlation†	Slope‡	Test§	NTBI comp||
All samples	0.61 (0.49–0.71),***			1.5 ± 2.59 μM vs 0.94 ± 1.13 μM,*
Transfused	0.57 (0.4–0.7),***	0.31 (0.21–0.41),***	ns, int**	2.04 ± 2.99 μM vs 0.76 ± 1.56 μM,***
Untrasfused	0.31 (0.02–0.55),*	0.83 (0.05–1.6),*		0.14 ± 0.71 μM vs -0.83 ± 1.91 μM,**
High sTfR	0.63 (0.48–0.74),***	0.25 (0.18–0.31),***	*	1.8 ± 2.9 μM vs 1.1 ± 1.16 μM,*
Low-normal sTfR	0.6 (0.28–0.8),**	0.83 (0.36–1.29),**		0.44 ± 0.83 μM vs 0.7 ± 1.13 μM, ns
Splenectomy	0.5 (0.24–0.69),**	0.14 (0.06–0.21),**	***	2.97 ± 3.7 μM vs 1.26 ± 1.02 μM,**
No splenectomy	0.68 (0.55–0.79),***	0.44 (0.33–0.54),***		0.84 ± 1.7 μM vs 0.73 ± 1.1 μM, ns
SF > 1500μg/L	0.55 (0.32–0.72),***	0.17 (0.1–0.25),***	*	0.82 ± 1.66 μM vs 0.71 ± 1.03 μM, ns
SF < 1500μg/L	0.65 (0.47–0.78),***	0.41 (0.280.54),***		2.83 ± 3.45 μM vs 1.49 ± 1.07 μM,*
LIC>12	0.77 (0.48–0.91),***	1.05 (0.6–1.49),***	*	0.45 ± 0.96 μM vs 0.86 ± 1.31 μM, ns
LIC<12	0.66 (0.24–0.87),*	0.42 (0.14–0.69),*		0.63 ± 1.67 μM vs 1.19 ± 1.06 μM, ns

*Abbreviations: ns*, not significant; *LIC*, liver iron content; *SF*, serum ferritin; *sTfR*, soluble transferrin receptors; *NTBI*, nontransferrin-bound iron.

*P*-value <0.0001***, <0.001**, <0.01*.

∗ Conditions where bead-NTBI and NTA-NTBI were compared.

† Pearson correlation coefficient (95% CI), *P* value.

‡ Slope (95% CI), *P* value.

§ Comparison of slopes *P*-value or when ns, the intercept (int) comparison *P*-value.

|| Mean NTBI comparison CP851 vs NTA (95% CI), *t* test p.

**Table I:** Patient diagnoses, patient number correlation within diagnoses, and NTBI differences for groups with n > 5

Diagnosis	n	Correlations∗	Grouped†	NTBI comparison‡
Eβ-thalassemia (Eβ-thal)	44	0.68 (0.48–0.81)***	n = 84, r = 0.63	1.3 vs 1.0***
β-thalassemia major (β-TM)	24	0.72 (0.44–0.87)***	(0.49–0.74)***	0.84 vs 0.82**
Thalassemia intermedia (TI)	11	0.5 (−0.14 to 0.85) ns		−0.06 vs 1.54 ns
Bart's Hb	3	N/A		N/A
α-Thalassemia	2			
Hereditary HFE Hemochromatosis (HH)	9	0.64 (−0.04 to 0.92) ns	n = 38, r = 0.66	−0.04 vs −1.67 ns
Sickle cell disease (SCD)	6	0.54 (−0.36 to 0.92) ns	(0.47 to 0.79)***	−0.07 vs −2.11 ns
Congenital sideroblastic anamia (CSA)	5			
Myelodysplastic syndrome (MDS)	3			
Diamond-Blackfan anemia (DBA)	2			
Pyruvate kinase deficiency anemia	2			
Aceruloplasminemia	2			
Ferroportin disease	2			
Red cell aplasia	1	N/A		N/A
Sickle-E-β-thalassemia (SCD)	1			
Congenital dyserythropoietic anemia	1			
β-thalassemia-Hb Malay anemia	1			
Hemolytic anemia	1			
Spherocytosis	1			
Atransferrinemia	1			
Normal volunteers	13	0 (horizontal line)		−0.1 vs −2.75***

*Abbreviations: NTBI*, nontransferrin-bound iron; *ns*, not significant.

*P* value < 0.0001***, <0.001**, <0.01*.

∗ Spearman correlation coefficient between NTA-NTBI and bead-NTBI, (95% CI), *P* value.

† Correlation coefficient between NTA-NTBI and bead-NTBI, (95% CI), *P* value in all thalassemia and all nonthalassemia diagnoses.

‡ Comparison of median bead-NTBI vs NTA-NTBI, [uM], Wilcoxon test.

**Table II:** Multiple regression models for NTA-NTBI, CP851-NTBI, bias (CP851-NTA), and transferrin saturation

Model	NTA-NTBI	CP851-NTBI	Bias	TfSat
Adjusted r-square	0.75^∗∗∗^	0.26^∗∗∗^	0.24^∗∗∗^	0.42^∗∗∗^
n	101	120	117	100
*Constant*	−3.89_0.27_^∗∗∗^	−1.37_0.59_^∗^	2.16_0.62_^∗∗^	42.17_5.01_^∗∗∗^
*TfSat*	0.056_0.003_ (0.86)^∗∗∗^	0.028_0.007_ (0.3)^∗∗∗^	−0.026_0.007_ (−0.34)^∗∗∗^	
*Splenectomy yes* = *1*	0.51_0.2_ (0.13)^∗^	1.32_0.47_ (0.23)^∗∗^	0.86_0.41_ (0.18)^∗^	
*Thalassemia yes* = *1*				31.19_5.67_ (0.52)^∗∗∗^
*Eβ-thal yes* = *1*				11.6_4.75_ (0.21)^∗^
*MDS yes* = *1*				42.1_15.3_ (0.21)^∗∗^
*CSA yes* = *1*				42.5_10.4_ (0.33)^∗∗∗^
*DBA yes* = *1*				47.8_15.2_ (0.24)^∗∗^
*SF [ug/L]*				0.006_0.001_ (0.31)^∗∗∗^
*sTfR [nM]*	−0.003_0.001_ (−0.12)^∗^			
*Chelation yes* = *1*			1.25_0.45_ (0.27)^∗∗^	
*DFO + DFP yes* = *1*		2.29_0.8_ (0.23)^∗∗^	1.59_0.71_ (0.19)^∗^	
*DFO yes* = *1*				
*DFX yes* = *1*			−1.81_0.61_(−0.26)^∗∗^	
*Normal yes* = *1*			1.42_0.7_ (0.19)^∗^	

*Abbreviations: NTBI*, nontransferrin-bound iron; *NTA*, nitrilotriacetate; *TfSat*, transferrin saturation; *MDS*, myelodysplastic syndrome; *CSA*, congenital sideroblastic anemia; *DBA*, Diamodn-Blackfan anemia; *DFO*, deferoxamine; *DFP*, deferiprone; *DFX*, deferasirox.

An empty cell indicates that predictor was not significant in a particular model. Statistics for predictors (italics) are given as absolute regression coefficient, its standard deviation in subscript, adjusted regression coefficient in brackets, followed by the significance of the predictor (***<0.0001, **<0.001, *<0.05). All other predictors insignificant (LIC, cardiac T2*, SGOT, SGPT, Ret, NRBC, Plt, WBC, Hb, Hct, MCV, MCH, MCHC, bilirubin, transfusion). Multiple linear regression on SPSS version 22 was used.

## Appendix Subgroup Univariate Analysis

### NTBI by underlying diagnoses

The relationship between both assays was broadly similar across different patient diagnoses although median bead-nontransferrin-bound iron (NTBI) values were typically higher than nitrilotriacetate (NTA)-NTBI for Eβ-thal, β-TM, SCD, HH, and healthy controls (Fig S1, Table I). Significant correlations but poor agreement between both NTBI methods were seen for Eβ-thal, β-TM, and all thalassemias, with negative values by both methods treated as zero (Fig S1, Table SI). Other diagnoses were not sufficiently represented to draw correlation or agreement conclusions, however in nonthalassemic group as a whole the correlation was significant (Table I). Normal control samples neither correlated nor agreed between the 2 assays but their bias could be corrected for (Fig S2*L*).

### Transfusion

In transfused patients, both the methods correlated but nontransfused patients correlation was weak. Slopes were significant in both the groups, but only intercepts differed (Table SI). Significant effect of transfusion seems to be limited to high TfSat as only samples with TfSat>90% show significant positive slope among transfused patients (Fig S3). Bead-NTBI was higher than NTA-NTBI in transfused and untransfused patients (Table SI).

### Erythropoiesis

High- and low-erythropoietic states were judged by soluble transferrin receptor (sTfR) levels above (high) and below (low-normal) 28.1 nM (ULN). NTBI methods correlated, the slopes were significantly positive but differed from one another, for high- and low-normal erythropoietic states. The NTA assay detected less NTBI relative to the bead assay for patients with high erythropoiesis compared with those with low-normal levels of erythropoiesis (Table SI, Fig S4*A*). Because a high-erythropoietic rate will increase iron clearance from transferrin, this is predicted to free up iron binding sites on transferrin thus increasing iron shuttling onto these sites and hence lower levels with the NTA assay.

### Splenectomy

In patients with and without splenectomy, the methods correlated well with positive slopes that differed significantly: bead-NTBI being higher, and the difference between methods being greater in splenectomized than unsplenectomized patient (Fig S4*B*, Table SI).

### Storage iron

For high- and low-ferritin patient groups (above or below 1500 μg/L), methods correlated well with significantly different positive slopes. There was greater discrepancy between NTBI levels in the high-ferritin than the low-ferritin group (Fig S4*C*, Table SI): for similar CP851-NTBI values, NTA method gives lower NTBI values in the former and higher in the latter group above about 2-μM bead-NTBI threshold. Methods were also compared in liver iron content (LIC) groups below and above 12 mg/gdw (SF = 1500μg/L corresponds in our study to LIC = 12 mg/gdw) showing medium correlation and significantly different positive slopes. In high LIC group, discrepancy between methods was greater (Fig S4*D*, Table SI). NTBI differences were insignificant on direct comparison: not all patients had LIC available (n = 35 vs n = 109 for ferritin), so comparison may be underpowered to detect differences. Taken together, these findings show that at high-storage iron (LIC or SF) the extent to which bead-NTBI values exceed NTA-NTBI is increased.

## References

[1] Hershko C., Graham G., Bates G.W., Rachmilewitz E. (1978). Non-specific serum iron in thalassaemia: an abnormal serum iron fraction of potential toxicity. Br J Haematol.

[2] Porter J.B., Abeysinghe R.D., Marshall L., Hider R.C., Singh S. (1996). Kinetics of removal and reappearance of non-transferrin-bound plasma iron with deferoxamine therapy. Blood.

[3] Gosriwatana I., Loreal O., Lu S., Brissot P., Porter J., Hider R.C. (1999). Quantification of non-transferrin-bound iron in the presence of unsaturated transferrin. Anal Biochem.

[4] Loréal O., Gosriwatana I., Guyader D., Porter J., Brissot P., Hider R.C. (2000). Determination of non-transferrin-bound iron in genetic hemochromatosis using a new HPLC-based method. J Hepatol.

[5] Porter J.B., Walter P.B., Neumayr L.D. (2014). Mechanisms of plasma non-transferrin bound iron generation: insights from comparing transfused Diamond Blackfan anaemia with sickle cell and thalassaemia patients. Br J Haematol.

[6] Brissot P., Wright T.L., Ma W.L., Weisiger R.A. (1985). Efficient clearance of non-transferrin-bound iron by rat liver. Implications for hepatic iron loading in iron overload states. J Clin Invest.

[7] Wang C.Y., Knutson M.D. (2013). Hepatocyte divalent metal-ion transporter-1 is dispensable for hepatic iron accumulation and non-transferrin-bound iron uptake in mice. Hepatology.

[8] Jenkitkasemwong S., Wang C.Y., Coffey R. (2015). SLC39A14 is required for the development of hepatocellular iron overload in murine models of hereditary hemochromatosis. Cell Metab.

[9] Bergeron C., Kovacs K. (1978). Pituitary siderosis. A histologic, immunocytologic, and ultrastructural study. Am J Pathol.

[10] Simpson R.J., Konijn A.M., Lombard M., Raja K.B., Salisbury J.R., Peters T.J. (1993). Tissue iron loading and histopathological changes in hypotransferrinaemic mice. J Pathol.

[11] Oudit G.Y., Sun H., Trivieri M.G. (2003). L-type Ca^2+^ channels provide a major pathway for iron entry into cardiomyocytes in iron-overload cardiomyopathy. Nat Med.

[12] Oudit G.Y., Trivieri M.G., Khaper N., Liu P.P., Backx P.H. (2006). Role of L-type Ca2+ channels in iron transport and iron-overload cardiomyopathy. J Mol Med (Berl).

[13] Evans R.W., Rafique R., Zarea A. (2008). Nature of non-transferrin-bound iron: studies on iron citrate complexes and thalassemic sera. J Biol Inorg Chem.

[14] Silva A.M., Kong X., Parkin M.C., Cammack R., Hider R.C. (2009). Iron(III) citrate speciation in aqueous solution. Dalton Trans.

[15] Silva A.M., Hider R.C. (2009). Influence of non-enzymatic post-translation modifications on the ability of human serum albumin to bind iron. Implications for non-transferrin-bound iron speciation. Biochim Biophys Acta.

[16] Hider R.C., Silva M.N., Podinovskaia M., Ma Y. (2010). Monitoring the efficiency of iron chelation therapy: the potential of nontransferrin-bound iron. Ann N Y Acad Sci.

[17] Aydinok Y., Evans P., Manz C.Y., Porter J.B. (2012). Timed non-transferrin bound iron determinations probe the origin of chelatable iron pools during deferiprone regimens and predict chelation response. Haematologica.

[18] de Swart L., Hendriks J.C., van der Vorm L.N. (2016). Second international round robin for the quantification of serum non-transferrin-bound iron and labile plasma iron in patients with iron-overload disorders. Haematologica.

[19] Singh S., Hider R.C., Porter J.B. (1990). A direct method for quantification of non-transferrin-bound iron. Anal Biochem.

[20] Esposito B.P., Breuer W., Sirankapracha P., Pootrakul P., Hershko C., Cabantchik Z.I. (2003). Labile plasma iron in iron overload: redox activity and susceptibility to chelation. Blood.

[21] Breuer W., Ermers M.J., Pootrakul P., Abramov A., Hershko C., Cabantchik Z.I. (2001). Desferrioxamine-chelatable iron, a component of serum non-transferrin-bound iron, used for assessing chelation therapy. Blood.

[22] Breuer W., Cabantchik Z.I. (2001). A fluorescence-based one-step assay for serum non-transferrin-bound iron. Anal Biochem.

[23] Ma Y., Podinovskaia M., Evans P.J. (2014). A novel method for non-transferrin-bound iron quantification by chelatable fluorescent beads based on flow cytometry. Biochem J.

[24] Evans R.W., Williams J. (1980). The electrophoresis of transferrins in urea/polyacrylamide gels. Biochem J.

[25] Garbowski M.W., Carpenter J.P., Smith G. (2014). Biopsy-based calibration of T2* magnetic resonance for estimation of liver iron concentration and comparison with R2 Ferriscan. J Cardiovasc Magn Reson.

[26] St. Pierre T.G., Clark P.R., Chua-anusorn W. (2005). Noninvasive measurement and imaging of liver iron concentrations using proton magnetic resonance. Blood.

[27] Carpenter J.-P., He T., Kirk P. (2011). On T2* magnetic resonance and cardiac iron. Circulation.

[28] Bland J.M., Altman D.G. (1999). Measuring agreement in method comparison studies. Stat Methods Med Res.

[29] Breuer W., Ronson A., Slotki I.N., Abramov A., Hershko C., Cabantchik Z.I. (2000). The assessment of serum nontransferrin-bound iron in chelation therapy and iron supplementation. Blood.

[30] Walter P.B., Macklin E.A., Porter J. (2008). Inflammation and oxidant-stress in beta-thalassemia patients treated with iron chelators deferasirox (ICL670) or deferoxamine: an ancillary study of the Novartis CICL670A0107 trial. Haematologica.

[31] Evans P., Kayyali R., Hider R.C., Eccleston J., Porter J.B. (2010). Mechanisms for the shuttling of plasma non-transferrin-bound iron (NTBI) onto deferoxamine by deferiprone. Transl Res.

[32] Srichairatanakool S., Kemp P., Porter J.B. (1997). Evidence for “shuttle” effect of NTBI onto desferrioxamine in thalassaemic plasma in the presence of NTA. Paper presented at: International Symposium: Iron in Biology and Medicine.

[33] Ito S., Ikuta K., Kato D. (2014). Non-transferrin-bound iron assay system utilizing a conventional automated analyzer. Clin Chim Acta.

[34] Makino T., Nakamura K., Takahara K. (2014). Potential problems in the determination of serum non-transferrin-bound iron using nitrilotriacetic acid and ultrafiltration. Clin Chim Acta.

[35] Huebers H.A., Finch C. (1987). The physiology of transferrin and transferrin receptors. Physiol Rev.

[36] Taher A., Musallam K.M., El Rassi F. (2009). Levels of non-transferrin-bound iron as an index of iron overload in patients with thalassaemia intermedia. Br J Haematol.

[37] Aydinok Y., Bayraktaroglu S., Yildiz D., Alper H. (2011). Myocardial iron loading in patients with thalassemia major in Turkey and the potential role of splenectomy in myocardial siderosis. J Pediatr Hematol Oncol.

[38] Garbowski M.W., Evans P., Porter J.B. (2015). Residual erythropoiesis protects against cardiac iron loading in transfusion dependent thalassaemia (TDT) by lowering labile plasma iron (LPI) through transient apotransferrin generation. Blood.

